# Design and implementation of a web-based platform for biodiversity valorization data management: from process modeling to user-centred evaluation

**DOI:** 10.1093/database/baag052

**Published:** 2026-09-11

**Authors:** Beatrice Amico, Carlo Combi, Mauro Commisso, Stefano Negri, Flavia Guzzo

**Affiliations:** Department of Computer Science, University of Verona, Str. Le Grazie 15, Verona, Italy; Department of Computer Science, University of Verona, Str. Le Grazie 15, Verona, Italy; Department of Biotechnology, University of Verona, Str. Le Grazie 15, Verona, Italy; Department of Biotechnology, University of Verona, Str. Le Grazie 15, Verona, Italy; Department of Biotechnology, University of Verona, Str. Le Grazie 15, Verona, Italy

## Abstract

Research aimed to valorize biodiversity, such as studies based on phytochemical characterization and bioactivity assessment, produces wide and heterogeneous datasets in which data collection is often non-standardized. This paper presents the design and implementation of a web-based plant metabolite database platform specifically developed to support biodiversity valorization research within the activities of the National Biodiversity Future Center, with a focus on the analysis of the Italian flora. The platform was designed to manage plant species, biological samples, experimental metabolites, molecular information, bioactivity data, and spectrometry results within an integrated and structured data environment. The system development followed an advanced, agile, and process-oriented methodology with strong and continuous involvement of biologists (i.e., the final users), from the collection and analysis of process- and data-related requirements to database modeling, considering the integration of existing external public databases. Following this initial phase, the development of the user interface proceeded, enabling researchers to insert data, perform advanced queries, and analyze and visualize spectral data through a dedicated tool. The web application was evaluated using the Questionnaire for User Interaction Satisfaction, which received high ratings from domain experts. This work demonstrates how the integration of process modeling and database design can enhance the reproducibility and standardization of research aimed at the valorization of biodiversity.

## Introduction

Throughout their evolutionary history, plants have developed diverse physiological adaptations and evolved a wide range of specialized metabolites that function as chemical mechanisms of adaptation and response to environmental and biotic or abiotic stresses, enabling them to persist across heterogeneous environments and interact with other organisms. *Biodiversity* refers to the complex of genes, species, and ecosystems of a given area [1], and preserving it is a priority in many countries, as it is fundamental for maintaining global earth health and providing a sustainable and stable ecosystem for human societies.

Among the worldwide initiatives for biodiversity preservation [2–4], the National Biodiversity Future Center (NBFC) [5] is the first Italian National Research and Innovation Center dedicated to biodiversity, established with funding from the Ministry of University and Research through European Union resources under NextGenerationEU. The centre involves more than 2000 researchers from universities, research institutes, and companies, who conduct basic, applied, and innovation-oriented research focused on Mediterranean biodiversity to develop effective strategies for monitoring, conserving, restoring, and valorizing species and habitats across Italian regions. The project selects ~700 plant species to assemble a core collection that proportionally represents all Italian plant families. Plant material is derived from botanic gardens, nurseries, and natural populations across different Italian regions.

According to this wide context, biodiversity-related research generates large amounts of data that are often collected and stored in non-standardized databases. Managing this data can be challenging because it requires both (i) the development of a complex database system able to manage the richness and heterogeneity of field, sample, species, and experimental data and (ii) support for research activities based on a deep understanding of the processes adopted by scientists, from sample collection to experimental and data analysis. Therefore, developing biological data management systems that explicitly support biodiversity-related research processes can help research scientists during their studies. To this end, structured database systems and specialized process-oriented software solutions enable researchers to systematically store, retrieve, and analyze metabolite data, thus enhancing research efficiency and ensuring data integrity.

To support biodiversity research, and specifically that performed within the NBFC project, we describe in this paper a web-based solution to (i) record new data collected during the experiments using a system that ensures interoperability, integrity, and avoids redundancy; (ii) connect and interact with existing public biological/chemical databases, to integrate different sources of information; (iii) graphically display the content of the database, giving the possibility to interact with experimental data through several user-oriented queries; (iv) analyse and integrate also spectrometry data.

**Figure 1:**
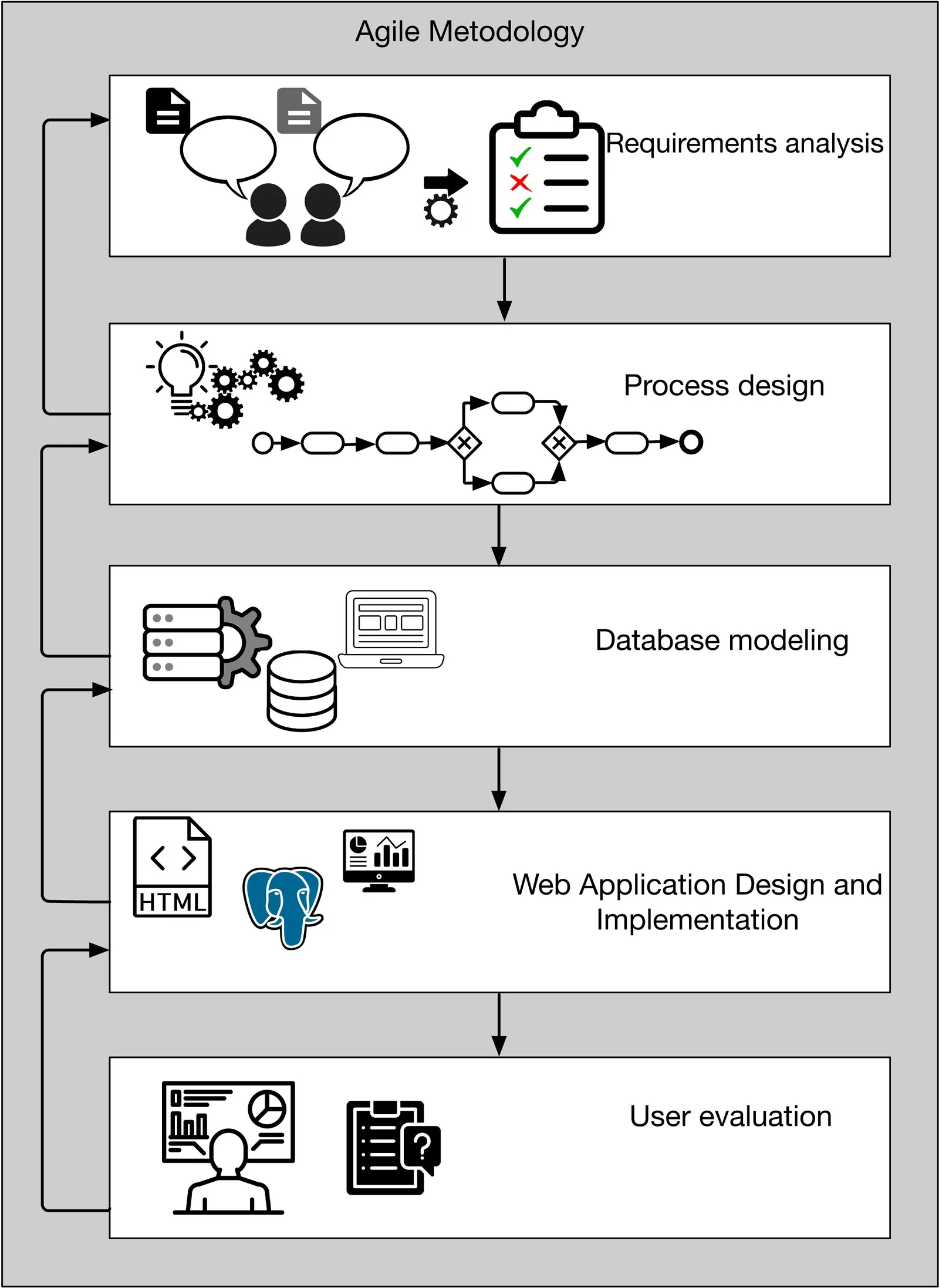
General overview of the proposed methodology.

While several plant-related databases have been recently proposed in the literature for different, specific purposes [6–10], none of them has been proposed to support biodiversity-related research in an integrated way. Moreover, while such contributions explicitly focus on plant data analysis, both for interactive query definition and for the visual representation of query results, none of them discusses the design process or the user evaluation of the proposed solutions. According to this scenario, the novelty of the proposed approach stems from the methodology proposed here, which seamlessly integrates different advanced development aspects. Indeed, the system is built on an agile and process-oriented methodology, composed of a series of steps and design/implementation tools, each contributing to the overall functionality. As we depict in Fig. 1, the methodology we propose starts by delineating the entire scientific workflow, from plant sampling to profiling and data analysis, formally represented using the Business Process Model and Notation (BPMN) [11], as discussed in the ‘Process modeling for Biodiversity’ section. This allowed us to graphically represent processes in a clear and expressive manner, making it accessible not only to domain experts but also to final users, thus facilitating eﬀective communication and understanding of complex workflows. The following step deals with the database design, as discussed in the ‘Database design’ section. It starts with the conceptual data modeling, which considers all the information requirements identified earlier during the Business Process Model (BPM) process modeling phase. After defining the conceptual schema, we proceed with the logical and physical modeling. During the final phase, we develop a web application with a graphical user interface (GUI) that respects the requisites from the previous phases.

All these aspects are described in the following sections. ‘Background’ section provides some basic definitions from the biological context, which are helpful for better explaining web application development. In the ‘System modeling and design’ section, we delineate the system design from the prerequisites analysis to the integration of external databases. ‘Web application design and implementation’ section describes the web application implementation and some core functionalities. ‘Graphical user interface and evaluation’ section describes the GUI and the evaluation process.

## Background

To fully understand the context of the NBFC project, it is essential to define some key biological concepts and methodologies, which are fundamental to the research activities of biotechnologists and to the purpose and functionality of the web application developed to support them. Understanding biodiversity and its taxonomic characterization provides the foundation for exploring the biochemical diversity of organisms, where the study of metabolites through advanced analytical techniques such as liquid chromatography–mass spectrometry (LC-MS) enables the identification and characterization of bioactive compounds crucial for ecological and functional studies. Biodiversity is defined as the variability among living organisms from all sources. Specifically, plant biodiversity is also linked to metabolite diversity through the multiple chemical compounds plants produce for growth, defense, and environmental interaction. Taxonomic research allows us to quantify, compare, and understand biodiversity, as well as the evolutionary relationships among different species. Taxonomy is defined as the field of science dealing with description, identification, nomenclature, and classification. Taxonomic research generally entails determining if a particular set of populations, generally studied from specimens, is discrete enough from others to warrant its own taxonomic status, e.g. as a separate species, infraspecies (subspecies, variety, or form), or higher-ranked taxon [12]. A metabolite is any intermediate or end product of metabolism, which encompasses all the physical and chemical processes involved in the maintenance and reproduction of life. During these processes, nutrients are broken down to generate energy and form simpler molecules (catabolism), which in turn can be used to synthesize more complex compounds (anabolism) [13]. To study bioactive compounds and metabolites, one of the analytical techniques is the LC-MS. This technique combines the separation power of liquid chromatography (or HPLC) with the mass analysis capability of MS. This method is highly sensitive and selective, making it suitable for a wide range of applications. LC-MS enables the separation, detection, and potential identification of chemical compounds with specific masses within complex mixtures, such as natural products from plant extracts or pure substances derived from mixtures of chemical intermediates [14]. Moreover, before entering into details of discussing the development of the web application, it is crucial to outline the primary data sources that supported this project and the methodologies used to structure and integrate this information into the database. To improve the completeness and consistency of the data, the system also integrated external resources, particularly PubChem [15] for chemical data and NCBI Taxonomy [16] for species classification. PubChem is a publicly accessible chemical database from the U.S. National Institutes of Health. It collects information on chemical structures, identifiers, chemical and physical properties, biological activities, patents, health, safety, and toxicity data, representing a useful source for the biodiversity research field. The integration of PubChem into the web application allows us to cross-reference experimental metabolites with their corresponding entries in PubChem through the compound ID. Likewise, the integration of taxonomic information is essential for organizing and classifying the biological species. Especially, the NCBI Taxonomy Database served as the primary source for taxonomic data.

Although these external resources provide taxonomic and chemical information, they do not manage the complete experimental context generated within the NBFC workflow. For example, PubChem provides compound-level chemical and bioactivity information, while NCBI Taxonomy provides standardized taxonomic classification; however, neither resource is designed to store project-specific sampling events, biological replicates, LC-MS experimental settings, user-curated metabolite annotations, and spectrometry outputs in an integrated operational environment. This gap motivated the development of a dedicated platform able to combine external reference data with newly generated experimental data.

Moving to consider plant databases, in the recent literature, some systems have been proposed, allowing the storage and the analysis of plant data for different purposes, e.g. understanding molecular mechanisms regulating plant key traits and biological processes through plant chromatin accessibility data [8]; finding the best medicinal plants to treat human diseases [10]; facilitating studies related to the regulation of plant lipid metabolism [6]; identifying the role of plant introns in plant growth and development [9]; analysing dispersed repeats in plant genomes [17]; and doing rapid analysis of plant gene families [7].

While these plant databases have been specifically designed for different purposes, they share some common features with our proposal (e.g., a web-based user interface, an underlying structured/semistructured database, and a query system). However, to the best of our knowledge, none of them is devoted to biodiversity research, offering a solution that spans from supporting experimental data collection to executing simple and complex user queries, through integration with external data banks.

## System modeling and design

In this section, we describe the design and the implementation of a relational database to manage biodiversity data on Italian flora species. We start with a requirements analysis modeled with Business Process Notation. After that, we focus on the database modeling and the integration with external databases.

The core of the system is a web-based application that allows users to execute queries, visualize and edit records, integrate new experimental results with external datasets, and perform different analyses. This platform provides biotechnologists with a specialized operational tool designed to support metabolomics research. It ensures the reproducibility of results, efficient storage and retrieval of data, and the integrity of biodiversity and metabolomics data stored on the platform.

The development process adopts a workflow-based approach to create software that accurately represents real laboratory practices. We began by analyzing the complete research lifecycle—from sample preparation to metabolite identification—systematically documenting each stage of data acquisition, processing, and interpretation. This analysis had two purposes: to capture the complexity of both biological and logistical aspects of research, and to identify bottlenecks, redundancies, and opportunities for automation. This approach provided the basis for process optimization, guided the integration of automated components, and ensured that the platform remained adaptable to evolving research needs. The design goal was not limited to static data storage but extended to create a dynamic system that supports throughput and reproducibility. System requirements were produced through stakeholder interviews to create a user-friendly interface, leading to the definition of key functionalities: how to store data, targeted queries with filtering options, the possibility to update records, and role-based access for authentication. We adopted an integrated process and information-modeling strategy to optimize the database schema, which accurately represents biological relationships, minimizing redundancy and enhancing data consistency.

### Process modeling for biodiversity

We formalized the workflows as BPM diagrams [11] using Camunda Modeler [18]. The adoption of BPM processes was motivated by the need to enhance the communication with biotechnology researchers, thereby supporting the collaborative development of the platform, enhancing process transparency, reproducibility, and automation in biological data curation.

The primary aim of this analysis was to gain an in-depth understanding of both the biological and logistical aspects of their activities, ensuring that the developed platform is fully aligned with their requirements. This alignment ensures that the digital platform reflects the operational realities of the researchers’ day-to-day practices and scientific objectives.

**Figure 2:**
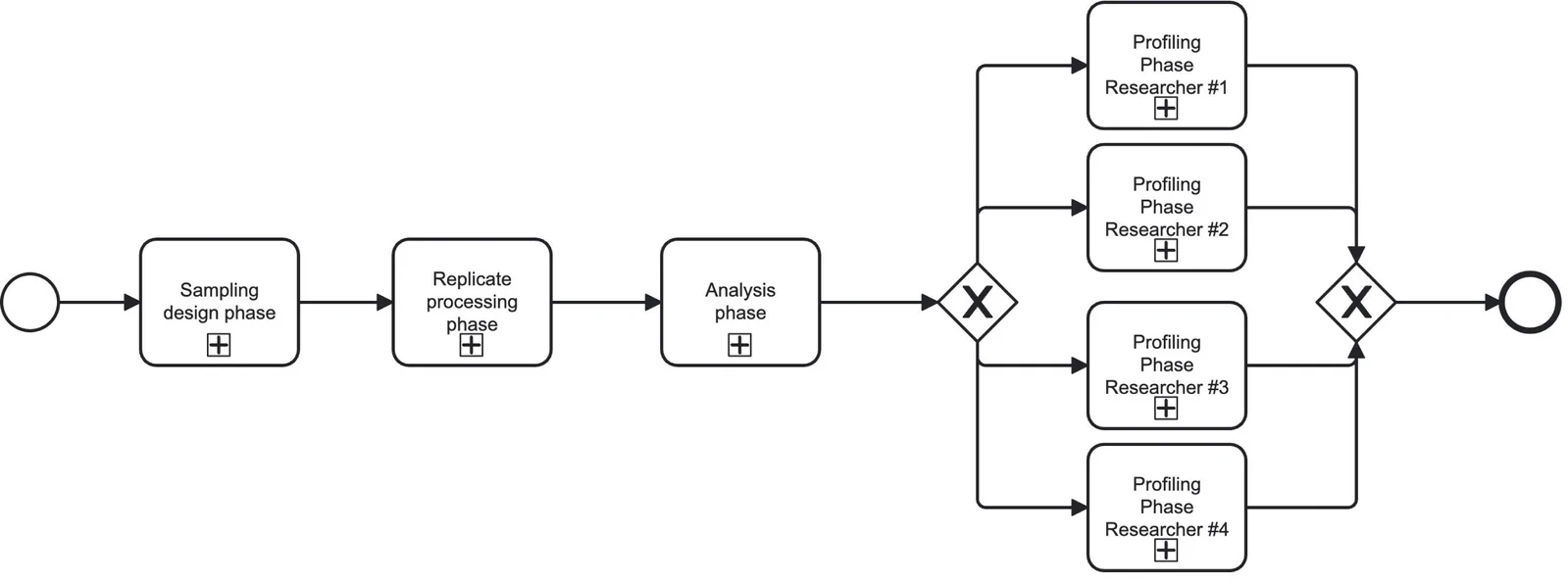
BPMN process of the entire framework of laboratory activities.

The workflow employed by biotechnologists at the University of Verona, represented in Fig. 2, is characterized by a well-defined sequence of activities. While the initial three stages—Sampling Design, Replicate Processing, and Analysis—are highly standardized and consistently applied by all researchers, the final phase, the Profiling ones, is characterized by a degree of individual customization to enhance efficiency and ease of interpretation.

#### Sampling design phase

**Figure 3:**
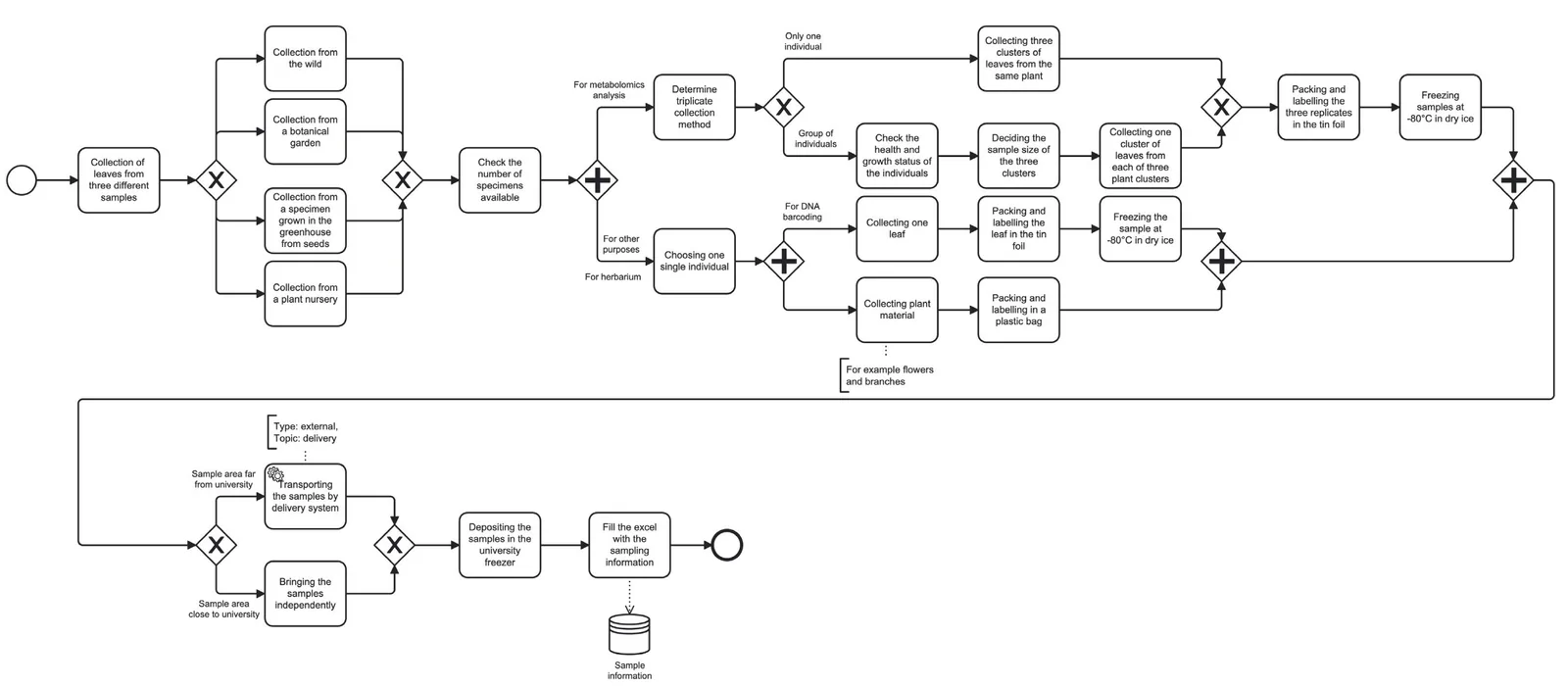
BPMN process for the sampling phase.

The initial step of the procedure, represented in Fig. 3, involves identifying a group of plants relevant to the sampling. This selection includes three different samples from three groups of plants, in order to ensure representativeness within the experimental design. When plant availability was limited, the three samples were collected from three distinct individual plants (number of plants per group = 1). Conversely, when only a single individual was available (e.g., a large tree in a botanical garden), three independent leaf samples were collected from the same individual. Depending on the analyzed plant, the workflow proceeds to the selection of a collection method. This selection includes (i) wild collection, which involves obtaining specimens directly from their natural ecosystems; (ii) botanical garden collection, where samples are maintained under controlled and well-documented conditions; (iii) greenhouse-grown specimen collection, using plants germinated from seed within university research facilities; (iv) plant nursery collection, involving acquisition of commercial plant material from certified suppliers.

These parallel collection pathways provide researchers with flexibility in sample acquisition while maintaining a consistent framework for documentation and quality control. The sampling process is based on a triplicate collection approach to ensure the reliability of experimental results. The choice of sampling strategy depends on the number of available plants and on the final application, which can fall into three broad categories: metabolomics analysis, requiring multiple samples from plant clusters for comprehensive profiling; DNA barcoding, where a single individual specimen is required for genetic identification; and herbarium preservation, aimed at the collection of plant material for long-term documentation. Two types of transportation are used depending on the location of the collection site: external transport systems are employed for samples collected from remote locations, whereas independent transport is used when samples are collected from nearby areas.

To prevent metabolic degradation and biochemical alteration, the samples were stored in polystyrene containers filled with dry ice until they arrived in the laboratory. After arriving at the university’s storage facility, the samples are frozen at −80°C. This rapid freezing ensures the integrity of the biochemical composition and protects the samples from deterioration prior to analysis. Finally, researchers record in an Excel spreadsheet all relevant sample information, including collection details, preservation methods, and final usage, to ensure proper traceability and compliance with data management protocols. For metabolomics analysis, the protocol involves either collecting three leaf clusters from a single plant, in cases of limited plant availability, or alternatively, one leaf cluster from each of three different groups of plants, with the number of plants per group ranging from 1 to 10 depending on plant availability. The samples are carefully labeled and wrapped in tin foil to preserve their integrity. For DNA barcoding, the procedure requires the collection of a single leaf from an individual plant specimen. For herbarium preservation, plant materials such as flowers or branches are collected and stored in plastic bags for subsequent archiving.

#### Replicates processing phase

**Figure 4:**
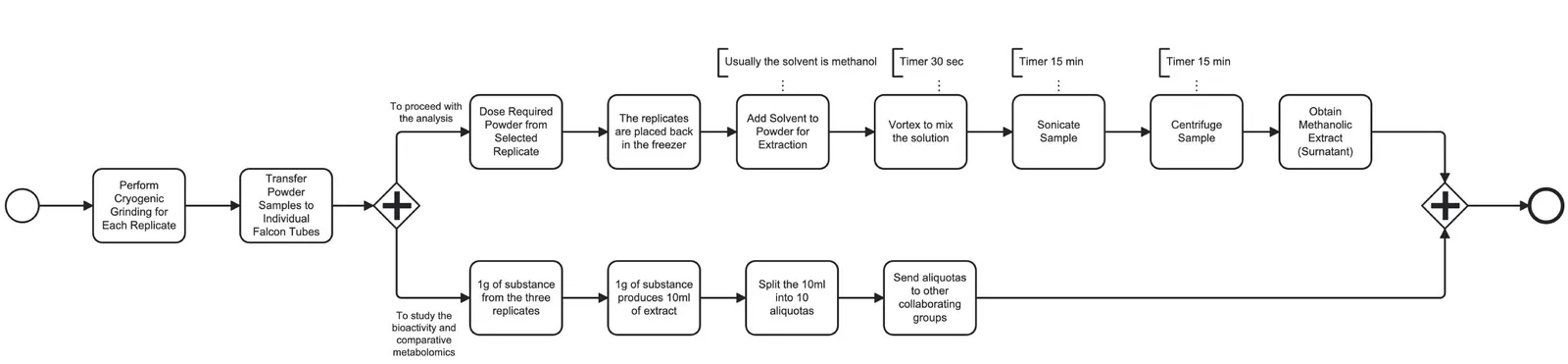
BPMN process for replicates processing phase.

This process, depicted in Fig. 4, consists of a series of operations designed to preserve the integrity of biological replicates and ensure the quality of the extracts. Initially, three biological replicates of the selected species, previously stored at –80°C, are retrieved and individually ground into a fine frozen powder using cryogenic grinding. The resulting powdered material from each replicate is subsequently subdivided into three aliquots, typically in sterile 15 ml Falcon tubes, thereby enabling parallel or future analyses and facilitating sample sharing with collaborating research groups.

For each replicate, a defined quantity of ground material (e.g., 1 g) is used to prepare a 10 ml extract. The obtained extract is further divided into 10 aliquots, which may be stored or distributed for collaborative purposes. The remaining ground material is retained for long-term storage, while one aliquot is subjected to solvent-based extraction.

Methanol is added to the sample, which is then vortexed for 30 s to enhance metabolite dispersion. The solution is sonicated for 15 min to provoke cellular disruption and facilitate molecular extraction. Furthermore, the mixture is centrifuged for 15 min to separate the extract molecules from the residual solid material. Finally, the supernatant, corresponding to the methanolic extract enriched in bioactive compounds, is carefully collected. This extract constitutes the purified molecular fraction used for subsequent analytical procedures, such as LC-MS profiling.

#### Analysis phase

**Figure 5:**
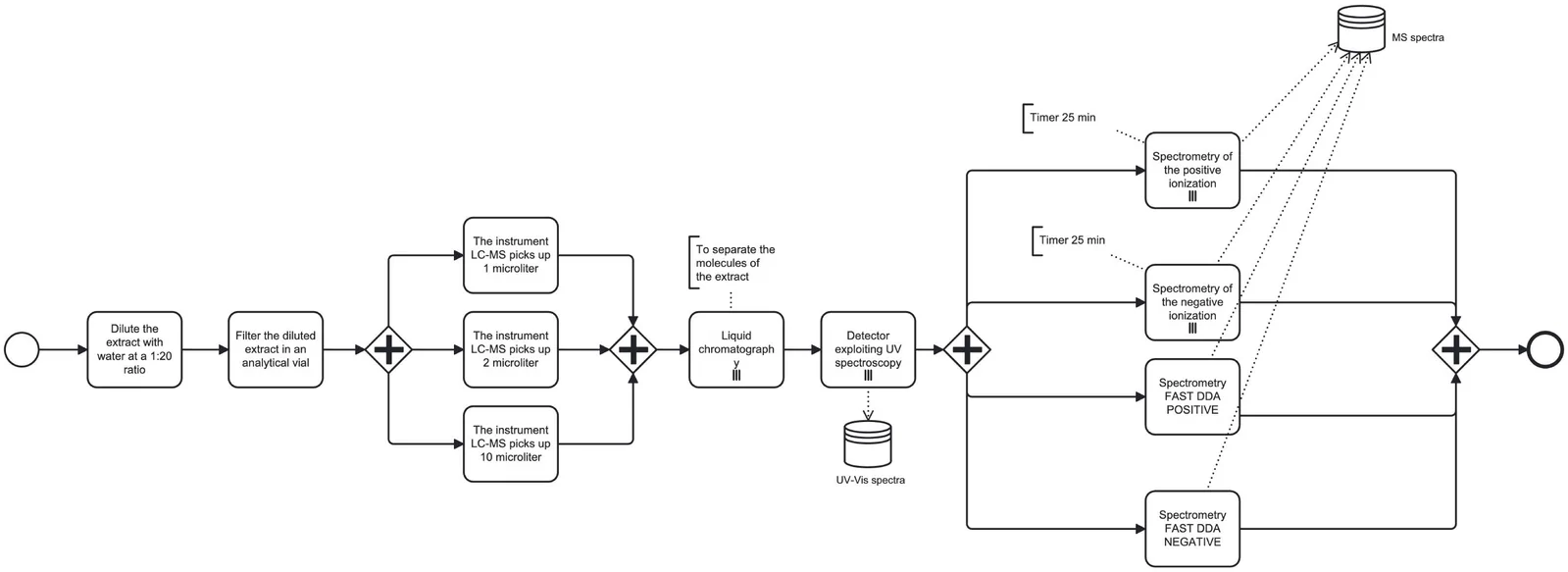
BPMN process for analysis phase.

This process, depicted in Fig. 5, is organized into several sequential stages, each contributing to the generation of high-quality spectral data for subsequent metabolomic interpretation. Initially, the extract is diluted with water in a proportion of 1:20. The diluted extract is filtered into an analytical vial to remove any particulate that could interfere with the next steps.

The LC-MS system takes 1, 2, or 10 µl from the diluted extract. Through a liquid chromatograph, molecules are separated according to their chemical properties. As the compounds elute, they are detected by a UV–vis spectroscopy detector, which records the corresponding absorption spectra. These spectra provide initial information on compound classification and preliminary identification.

Following UV/vis detection, the eluting compounds are subsequently introduced into a quadrupole time-of-flight mass spectrometer for mass analysis and detection. Finally, all UV–vis spectral data and MS outputs are compiled to form the analytical basis for downstream processes, including metabolite profiling, compound identification, and comparative analyses among biological samples.

#### Profiling

The metabolite profiling phase follows a common framework that can be outlined as (i) preparation of chromatographic data; (ii) processing and validation of chromatographic results; (iii) peak ranking; and (iv) analysis and documentation of results.

Despite this general structure, profiling strategies differ among researchers due to different experiences, methodological preferences, and analytical objectives. The analytical workflow typically begins with the acquisition and preprocessing of LC-MS data. This step involves data transformation procedures intended to enhance signal clarity, improve baseline stability, and increase overall reliability. The peak identification phase, relying on retention time (RT) and intensity parameters, is fundamental to ensure that only relevant metabolites are selected for further analysis. Researchers routinely query different external databases, including PubChem, the Human Metabolome Database, and MassBank, within each workflow. This comparison of mass-to-charge (*m*/*z*) ratios and fragmentation patterns enables the annotation of detected peaks with known metabolites. This is an important functionality supported by the requirements analysis. Finally, the profiling phase concludes with the systematic organization, filtering, and interpretation of the processed data, providing the foundation for subsequent metabolomic comparisons and biological interpretations.

### Database design

#### Conceptual modelling

After a detailed analysis of the workload through the use of BPMN processes, we proceed with the conceptual modeling. In order to design a database to manage this data, through conceptual modeling, we are able to capture and organize the complex and heterogeneous information arising from various domains—chemical, taxonomic, bioactivity, spectrometric, and geographic data—into a coherent and formal representation. In a close collaboration with biotechnologists, we first identified the relevant entities to capture the complexity of plant metabolomics data. Each entity corresponds to a fundamental biological concept—such as metabolites, plant taxonomy, sample registration, or experimental conditions—and is described by biologically meaningful properties that make the stored information both complete and interpretable. This approach provided additional insight into the structure of the data and the relationships between different biological concepts. The resulting entity–relationship (ER) model served as the foundation for designing the relational database schema, which formally specifies the database structure. By defining constraints such as unique identifiers, cardinalities, and associations, the conceptual model ensured data integrity and consistency.

**Figure 6:**
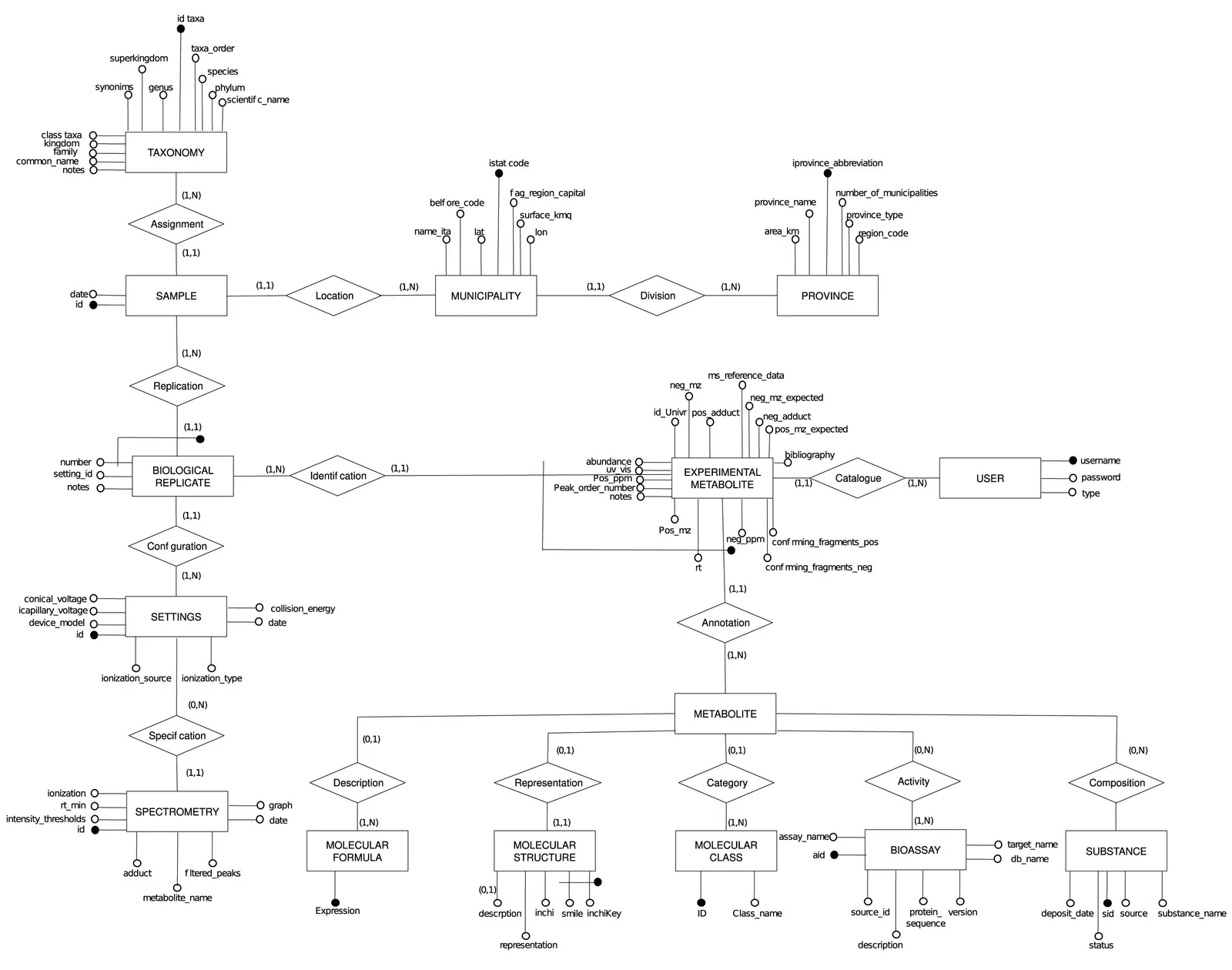
The ER schema of the overall database.

In Fig. 6, we present the ER model obtained after this phase. The model can be described as four main subject areas, each addressing a specific dimension of plant metabolomics data management.

The first area, Sampling and Taxonomy, captures information related to biological samples and their classification. The taxonomy entity describes the complete hierarchical structure (from kingdom down to species), taxonomic identifiers, and auxiliary metadata such as common names and synonyms. Together with the sample information, it ensures that the biological material is fully categorized both taxonomically and geographically.

The second area, Experimental Analysis, describes the experimental data generated during metabolomics analysis. The spectrometry entity stores MS outputs, including RTs, ionization methods, metabolite names, and filtered peak lists, while the experimental metabolite entity records detected spectral features, including *m*/*z* values, abundances, adducts, fragment confirmations, and bibliographic references.

The third area, Metabolites and Molecular Characterization, focuses on the chemical and biological description of metabolites. The metabolite entity provides detailed information on fundamental characteristics, including molecular formula, SMILES, InChI, InChIKey, molecular weight, PubChem Compound ID (CID), CAS number, and structural images (e.g. PNG or JPEG). Additionally, through links to PubChem BioAssay identifiers (AIDs), assay descriptions, and results, we record data of biological activity, enabling a connection between chemical structure and functional properties.

Finally, the Data Management area manages authentication credentials (username and password) and role definitions, supporting controlled access to the system and secure data management.

#### Logical and physical design

The transition from conceptual modeling to logical and then physical modeling involves defining the actual structure of tables, columns, data types, and integrity constraints. The logical model is implemented using SQL. Physical modeling ensures that the resulting database accurately reflects the original ER schema while being optimized for efficient data storage, retrieval, and management.

For example, in Listing 1, we present the table designed to store gold standard metabolite spectrometry data.

Listing 1: Spectrometry table in PostgreSQL.

CREATE TABLE public.spectrometry (

 id SERIAL

 PRIMARY KEY,

 intensity_threshold DOUBLE PRECISION,

 rt_min DOUBLE PRECISION,

 ionization DOUBLE PRECISION,

 adduct VARCHAR(50),

 graph BYTEA,

 filtered_peaks JSONB,

 date TIMESTAMP DEFAULT CURRENT_TIMESTAMP,

 metabolite_name VARCHAR(50),

 setting_id INTEGER

 CONSTRAINT spectrometry_setting_id_fk

 REFERENCES public.setting

);

ALTER TABLE public.spectrometry

 OWNER TO postgres;

### Integration with external DB

The integration of publicly available databases significantly strengthens the system’s analytical capabilities, providing biotechnologists with a more comprehensive and insightful tool for metabolomic research. Specifically, the system integrates two databases: NCBI Taxonomy [16] for taxonomic classification and PubChem [15] for chemical data. Integrating PubChem into the web application enabled the cross-referencing of experimental metabolites with their corresponding PubChem entries using unique identifiers, such as CIDs. This allowed the retrieval of additional chemical information—such as molecular formulas, molecular weights, and structural representations (SMILES or InChIKey)—that was not present in the original dataset. Moreover, the integration made it possible to connect metabolites with known biological activities and pharmacological properties, when available. Thus, the web application effectively bridges experimental data with existing chemical knowledge, allowing researchers to place their findings within the research context of chemical biology.

The integration of the NCBI Taxonomy Database allows for automatic retrieval of the complete taxonomic hierarchy (e.g., class, order, family) of a plant species, and enables the standardization of species names, minimizing ambiguity and improving data consistency.

## Web application design and implementation

### System architecture

In this section, we detail the system architecture from the backend to the front end, describing the different technologies we used, and then we describe some core functionalities such as the user definition, data insertion, and update queries. The objective was to develop a framework that would enhance researchers’ work, make it more eﬃcient, minimize duplicate work, and ensure that valuable research data are systematically stored and processed. The development of the web application adopted an Agile methodology due to the continuous interactions with the final stakeholders to develop a functional web application that can be used in real-world scenarios. The development phase began with a series of preliminary meetings aimed at producing a prototype that respects the final user’s requirements. During these meetings, the design process served as the foundation for defining how the data would be structured, accessed, and processed within the application. Within the project, we organized frequent collaborative meetings with the biotechnologists where different requirements were systematically elicited, from additional query functionalities to more sophisticated interface improvements. The backend was developed using Python due to its simplicity, readability, and extensive library support. Flask was adopted as a lightweight Web Server Gateway Interface web framework, providing a modular and scalable architecture. It managed HTTP routing, user authentication, and integration with the database. Jinja2, the templating engine associated with Flask, was used to render HTML pages, integrating data retrieved from the database in a structured manner. SQLAlchemy was adopted as an Object-Relational Mapper (ORM) to facilitate interactions with the PostgreSQL database, enabling object-oriented manipulation of data through the mapping of database tables to Python classes. In addition, the backend architecture was further reinforced through the utilization of additional Python libraries, Pandas for comprehensive data processing, and Requests for the management of external API calls.

For frontend development, the application was constructed using HTML, CSS, and JavaScript, with Bootstrap as the primary framework for styling and layout. While prebuilt components—such as navigation bars, forms, and modals—accelerated the development and maintained a professional appearance. JavaScript was utilized to enhance user interaction, supporting functionalities such as dynamic form validation, real-time content updates, calculations, and immediate feedback to user actions. Multiple content delivery networks were leveraged to deliver resources, including Bootstrap CSS and JavaScript, Bootstrap Icons, jQuery, Plotly.js for interactive visualizations, and Select2 for advanced dropdown menus.

The architecture of the web application adopts the Model–View–Controller pattern [19], a software engineering approach that decomposes an application into three interdependent components: Model, View, and Controller. The Model component is responsible for managing the application’s data and business logic. The View component is responsible for presenting data to the user and handling the user interface. The Controller component handles user requests, orchestrates the flow of data, and acts as the intermediary between the Model and the View. This is particularly effective for interactive web applications, as it supports multiple representations of data, promotes code reuse, and facilitates a modular focus on distinct functional aspects.

For the development and management of the NBFC database, we adopted PostgreSQL as the database management system, primarily due to its open-source nature and cost-free accessibility. pgAdmin provided an intuitive interface for the visualization of data structures, the database object manipulation, and the execution of SQL queries. It was employed for the implementation of the ER schema, enabling the translation of entities, attributes, and relationships into corresponding tables, primary keys, foreign keys, and constraints. With the use of these systems, we enhanced the efficiency of schema development and ensured structural coherence in the management of biochemical data.

### Core functionalities

During the evolution of the project, the ER schema was modified through a series of evolutions to accommodate new requirements, improve data integrity, and enhance query performance. To implement the integration of the relational database into the web application, we used SQLAlchemy, employed as the ORM.

#### User definition

After discussing with the stakeholders the different possible actions feasible in the application, we outlined three types of users:

- Superuser: this role is only for advanced users, such as the head of the project, a researcher with a high level of expertise, or a system administrator. They have permissions to enter new data, modify existing records, and perform all types of searches within the database.
- Guest: this role is for external researchers who have full access to the experimental data, but do not have authorization to insert or modify data.
- Visitor: this role is for sporadic access to the application. They have restricted access, limited to a predefined set of queries and to viewing specific information, such as basic metabolite research and taxonomy. Experimental data is not accessible.

Additionally, we integrated spectrometry data associated with gold standard metabolites and developed a dedicated spectrometry tool to analyze these data.

#### Data insertion and update queries

The web application plays a fundamental role in facilitating the insertion of new experimental metabolites and the modification of existing data. This feature, implemented to help biotechnologists during the metabolite profiling phase by allowing them to eﬀectively record and manage experimental results through the database, avoids duplicates and manual errors and respects constraints between tables. This functionality guarantees that all relevant data is stored in the database. Some of the functionalities are

- Insert a new experimental metabolite: This functionality allows users to directly insert into the database new experimental metabolites identified during the profiling phase;
- Update an experimental metabolite: The web application allows users to update specific fields of an experimental metabolite directly from the metabolite visualization page. These fields are the metabolite name, molecular formula, chemical class, and molecular weight. This functionality is also available for the experimental data fields, including peak ID, UV–vis values, expected, and experimental *m*/*z* values, confirming fragments, adducts, ppm values (both positive and negative), MSI level, and bibliographic references;
- Insert gold standard metabolite spectrometry data: The process starts with the upload of an Excel file generated by the MS instrument. This file contains a raw list of detected peaks, including *m*/*z* values and absolute intensities, and is typically analyzed manually by biotechnologists to select peaks above a certain intensity threshold. The web application automates this process by calculating the relative intensity (%BPI) based on the highest intensity in the dataset and allowing users to apply an intensity threshold to filter out less significant peaks. After this selection, the application generates an interactive spectrum graph, displaying *m*/*z* values on the *x*-axis and relative intensity on the *y*-axis. Researchers can manually select or deselect peaks to refine the final graph, which can be saved for future analysis. Users are also required to enter experimental parameters, including the metabolite name, RT, ionization mode (positive or negative), adduct type, and instrumental settings used during the LC-MS analysis. Once all the information has been validated, the data are saved to the database.

## Graphical user interface and evaluation

This section describes the user interface design, with particular attention to the different functionalities and the web application evaluation process.

### User interface design

The GUI was designed to adhere to the BPMN processes defined during the requirements analysis. The implementation process followed an iterative methodology in a close collaboration with domain experts. Through structured feedback sessions, we refined various prototypes to fulfill users’ requests.

At first, the system supported only basic queries, such as retrieving all metabolites associated with a given species or identifying the species linked to a specific metabolite. Subsequently, in response to user needs, the system was extended to produce more advanced querying functionalities, guiding the users through complex tasks such as data curation, metabolite quantification, and comparative profiling, thereby improving usability, minimizing potential errors, and ensuring data integrity. We developed a dedicated data entry interface to enable researchers to record experimental data directly into the system. The user interface was designed to reproduce the structure of an Excel template, used in their laboratory workflow. Another part of the system considers the management of gold standard metabolites—reference compounds analyzed under identical LC-MS conditions and employed to enrich the internal database. This module enables the structured entry and administration of such compounds, thereby strengthening the application’s role in long-term data curation and the standardization of metabolite information.

**Figure 7:**
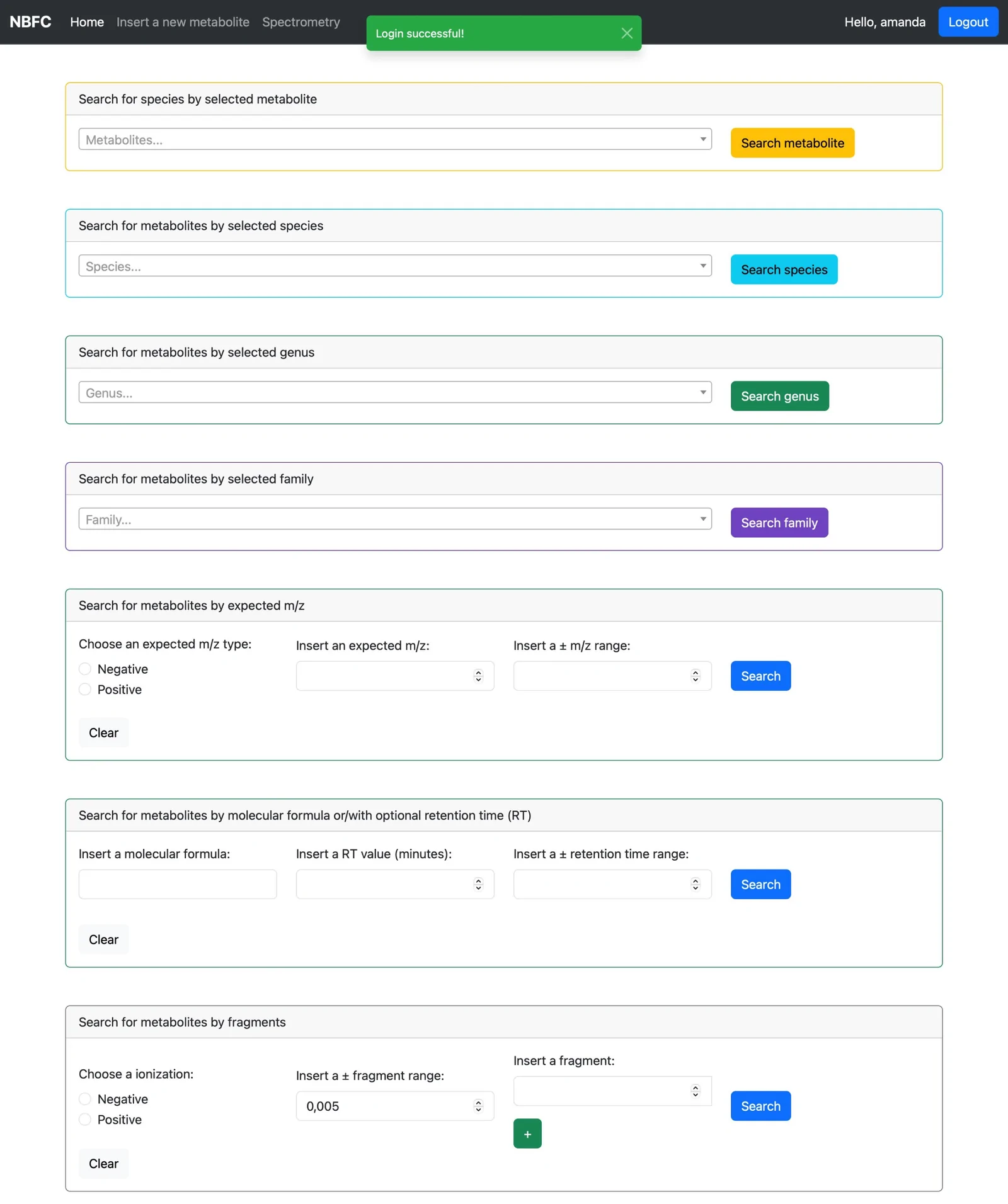
Homepage of the web application.

In Fig. 7, we report the web application’s homepage, the web page accessible after an authentication procedure. During the requirements analysis phase, the biotechnologists explicitly requested the implementation of a comprehensive homepage that would serve as an entry point to all the application’s functionalities. This page provides an overview of the available tools, enabling users to conveniently select the functionality they want to utilize.

**Figure 8:**
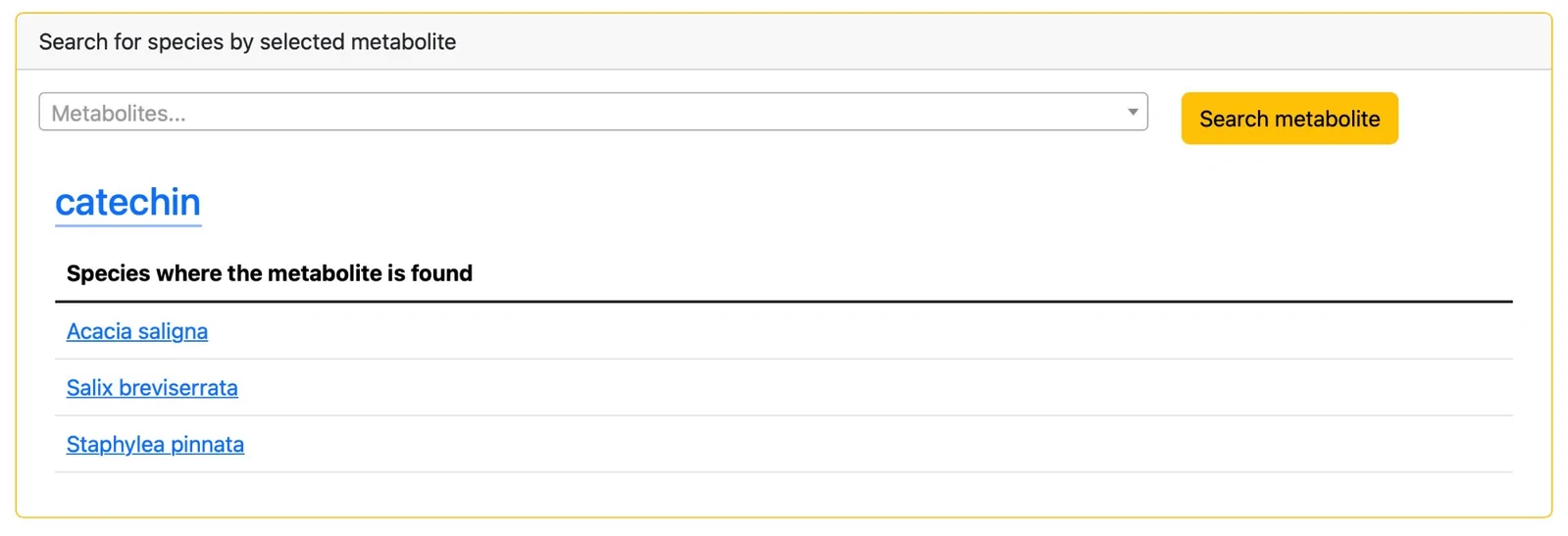
Search for Species by Selected Metabolite interface, showing results for the metabolite catechin.

**Figure 9:**
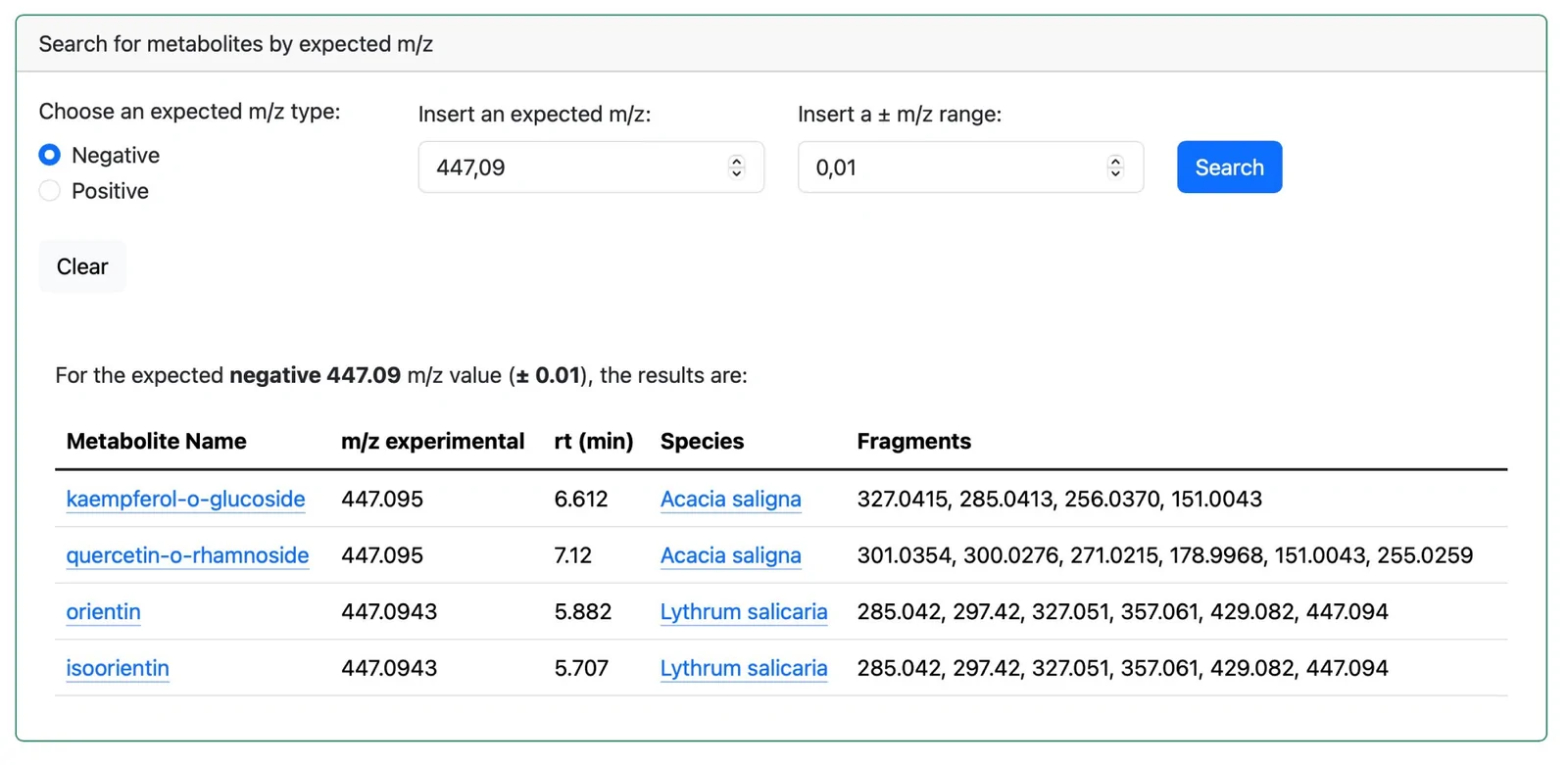
Search for Metabolites by Expected *m*/*z* interface.

The set of search and filtering options is composed as follows:

- Search for species by selected metabolite: Selecting a metabolite from the dropdown menu, the system retrieves the corresponding species. This list is automatically populated with all metabolites stored in the database. The name of the selected metabolite appears as a clickable link to the Metabolite Detail page. On this page, the user finds general information about the metabolite, including its chemical properties, and lists all the species in which it is found. For each species, we have a clickable link, leading the user to the Metabolite Species page.
- Search for metabolites by selected species: This search functionality allows users to explore the metabolites associated with a specific species. Even for the species, the dropdown list is associated with the species available in the database. Once a species is selected, the system retrieves all metabolites identified during the profiling phase of that species. The results are organized in a two-column table, where the first column lists all metabolites detected in the selected species, with each entry serving as a clickable link that redirects to the Metabolite Detail page. At the same time, the second column contains corresponding links to the Metabolite Species page. This design provides a concise and organized overview of the species metabolite, allowing users to focus exclusively on the information most relevant for their analyses. For example, as shown in Fig. 8, selecting the metabolite catechin returns the following species: *Acacia saligna, Salix breviserrata*, and *Staphylea pinnata*. Each of these species is linked to its own page, where users can access experimental data.
- Search for metabolites by selected genus: This functionality enables users to investigate metabolites of a specific genus, giving the user the possibility of studying the taxonomy hierarchy of these metabolites. By selecting a genus from the dropdown menu, the system dynamically retrieves the metabolites identified within the chosen taxonomic group. The results are reported as an interactive list of metabolites, each displayed as a clickable link that redirects to the Metabolite Detail page.
- Search for metabolites by selected family: The functionality for family follows the same structure as the genus-level search. It enables the users to retrieve metabolites associated with a selected family.
- Search for metabolites by expected *m*/*z*: This search functionality enables users to filter experimental metabolites according to the expected mass-to-charge ratio (*m*/*z*). During the profiling phase, it allows researchers to rapidly compare predicted or literature-reported *m*/*z* values with experimental data. Users have to select the ionization mode (positive or negative), an essential step to determine whether the system queries the database for positive or negative expected *m*/*z* values, with a range of ±0.01. Then the application provides the output as a list of metabolites that respect the specified criteria, displaying the list of metabolite names as a clickable link that redirects the user to the Metabolite Detail page, the experimental *m*/*z* values, the RTs, the species in which the metabolites are identified, and the associated fragment information. Figure 9 shows an example where the user specified an expected negative *m*/*z* value of 447.09 (±0.01), and the query returned four matching metabolites.
- Search for metabolites by molecular formula or/with optional RT: This functionality offers researchers a flexible tool for filtering experimental metabolites based on either a molecular formula, an RT within a specified range (default ±0.01 min), or a combination of both criteria. This functionality is useful when different metabolites share the same molecular formula, and the inclusion of RT facilitates the identification of isomers or structurally related compounds.
- Search for metabolites by fragments: This last search functionality enables users to identify metabolites based on their fragmentation components. During the profiling phase of a new metabolite, observing the fragmentation pattern can act as a molecular fingerprint. By comparing observed fragments with established fragmentation patterns, researchers can hypothesize potential molecular structures or confirm the identities of compounds found in biological samples.

Access to these features is regulated according to user roles: superusers and registered users are granted full access to all search options, whereas guest users are limited to the first four search types. This controlled access ensures that sensitive experimental data remain protected and accessible only to authorized users.

At the top of the page, we have a navigation banner that provides quick and intuitive access to the application’s main sections. The *Home* button allows users to refresh and return to the main page, while the *Insert a new metabolite* option is dedicated to the insertion of new experimental metabolite data. The *Spectrometry* button grants access to the page where it is possible to upload a gold standard and analyze metabolite spectrometry data.

**Figure 10:**
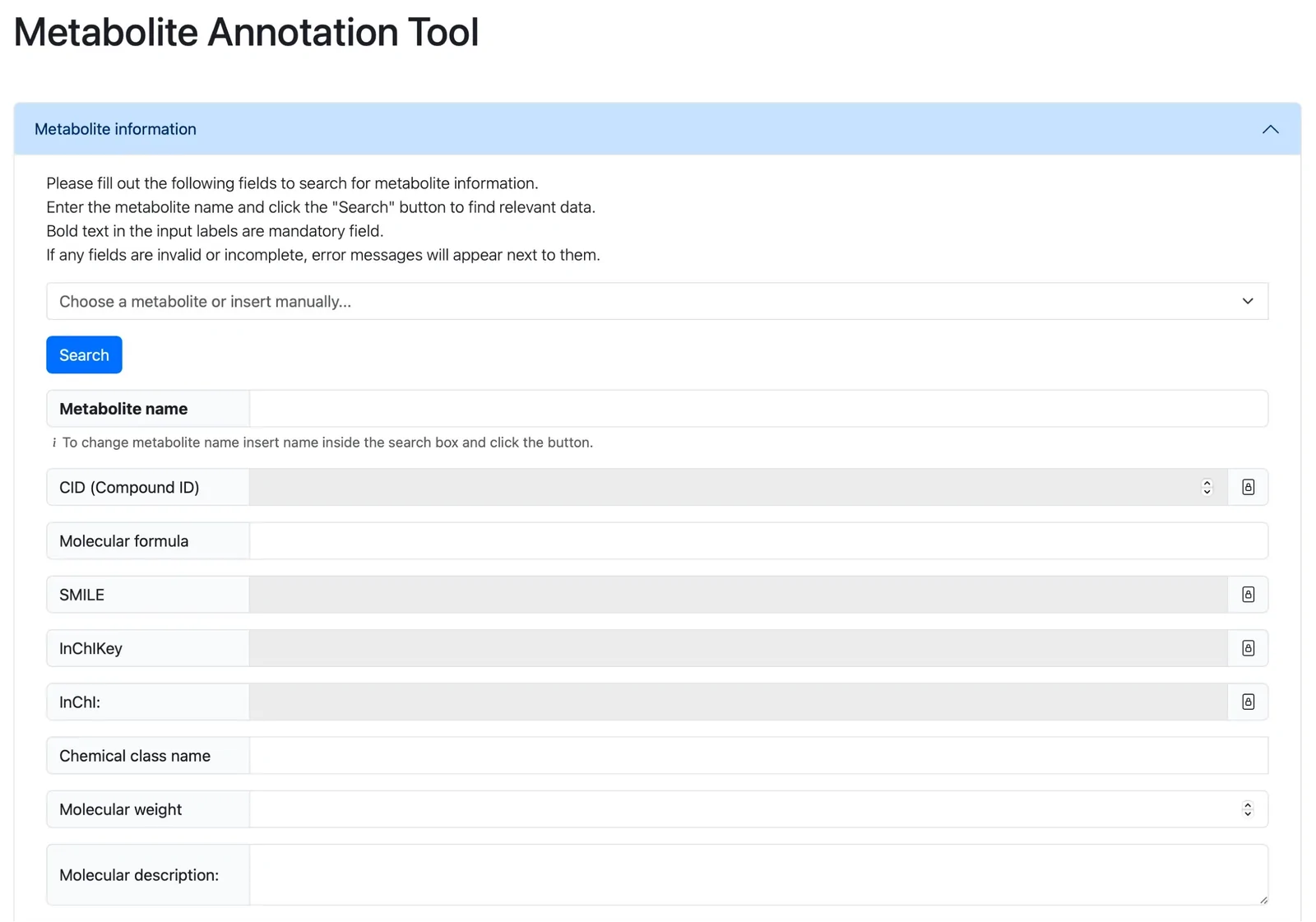
The Metabolite Information section, showing the interface for entering or auto-populating basic metabolite details.

Detailing the second tab, the ‘Insert a New Metabolite’ page is accessible only to users with superuser privileges. This page is divided into six distinct sections, each presented as a collapsible panel to enhance navigation. The sections include Metabolite Information, LC-MS, Taxonomy Information, Latest Insertions for the Selected Species, Sample and Biological Replicate, and Settings Information, as depicted in Figure 10. In the Metabolite Information section, users have two options for entering data. The first option allows the system to automatically fill in certain fields using existing information from the database. These fields include Molecular Formula, SMILE, InChIKey, InChI, Chemical Class Name, Molecular Weight, and Molecular Description.

Alternatively, users can manually input all the information, which will prompt the system to initiate an API query to PubChem to retrieve the relevant metadata. Of all the fields, only the metabolite name is mandatory. This design allows the system to accommodate the registration of new entities or compounds for which structural or descriptive data may still be incomplete.

**Figure 11:**
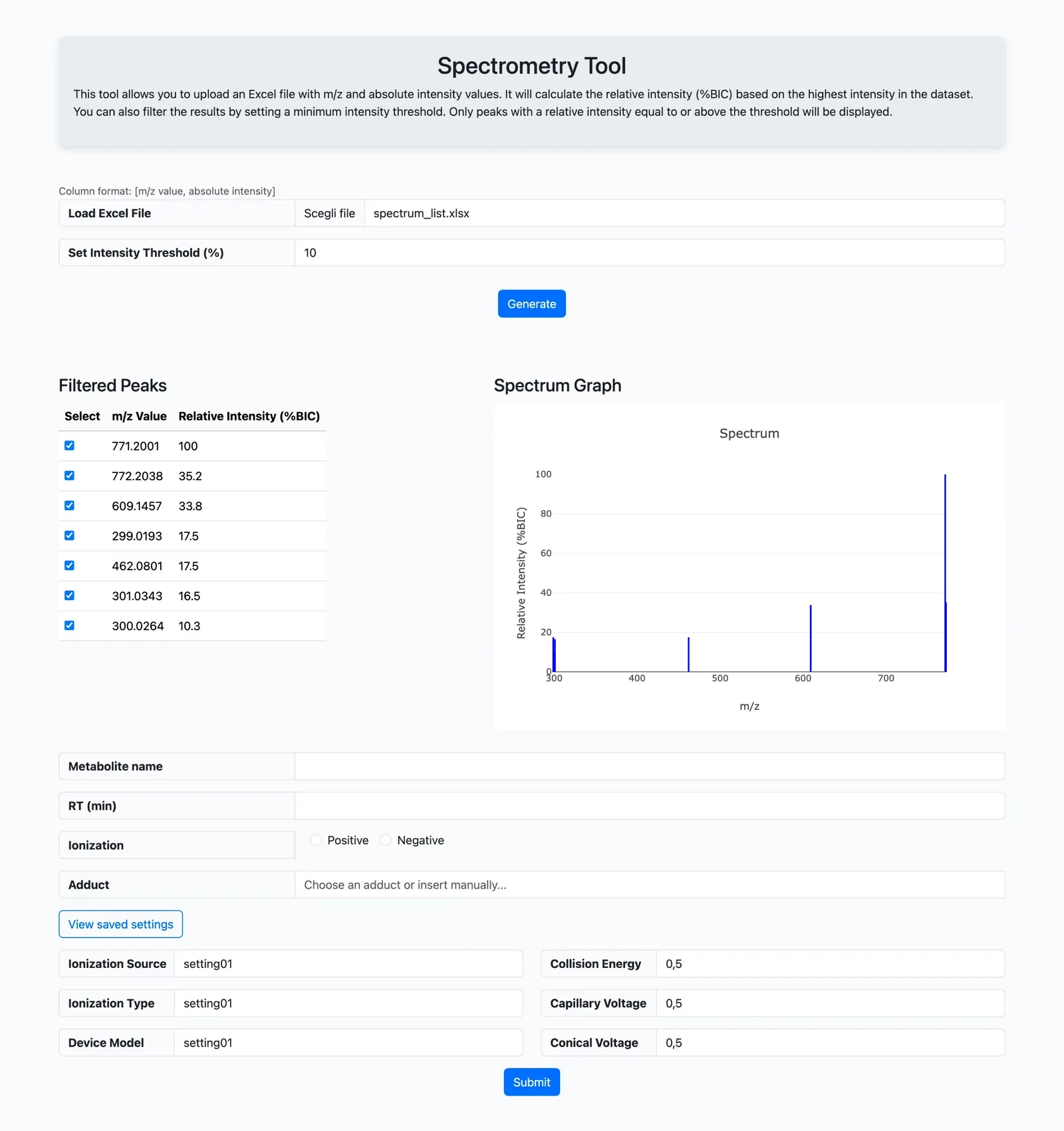
The Spectrometry page for uploading and analysing gold standard metabolites.

The spectrometry analysis plays a crucial role in plant biochemical profiling. Thus, in the web application, the LC-MS data have a dedicated section specifically designed for spectrum analysis. This functionality enables the user to visualize and interpret spectral data, performing an accurate characterization and comparison of plant metabolites. The process begins with the upload of an Excel file containing the *m*/*z* values and their corresponding absolute intensities. The application automatically calculates the Relative Intensity (%BIC) for each peak, using the highest intensity in the dataset as a reference. To deal with noise in the spectrometry data, the application provides an option to set an intensity threshold to automatically filter out low-intensity signals. As illustrated in Fig. 11, a threshold of 10 excludes all peaks with a %BIC below this value from the visualization. The final table includes columns for peak selection, *m*/*z* value, and %BIC. The application generates a spectrum plot that displays the selected peaks, with *m*/*z* values on the *x*-axis and %BIC on the *y*-axis. This plot is dynamic in response to user interactions. Thus, when the user toggles some peaks in the tables, the spectrum is refreshed. This functionality enables researchers to refine the spectral representation in an iterative manner.

### Web application evaluation

The evaluation process provided an opportunity for end users to actively interact with the system and offered practical feedback based on real use cases. We prepared a set of structured queries accompanied by detailed step-by-step instructions—beginning with the login procedure and guiding users through data exploration, entry, and query execution. For each test case, we recorded the execution time. The queries were related to different tasks associated with the features implemented in the platform, such as searching for species, selecting a specific metabolite, searching for metabolites, selecting a specific species, and searching for metabolites, selecting a specific genus. A selection of representative evaluation questions is reported below:

- Search for all species associated with the metabolite B-type procyanidin dimer and view all fragment information associated with this metabolite.
- Search for all metabolites associated with the species *S. pinnata*.
- Find all metabolites associated with the family Fabaceae.
- Open the Spectrometry section, upload the spectrum list file, complete the form, and save the data.

After this question-based evaluation phase, we administered the tailored Questionnaire for User Interaction Satisfaction (QUIS), a well-established tool for measuring user satisfaction with human–computer interfaces [20], to three researchers from the NBFC project, all sharing a background in plant metabolomics, but representing different levels of seniority. The questionnaire was tailored to assess aspects such as ease of use, intuitiveness, and eﬃciency of the interface, as well as overall satisfaction with the application. Each item was rated on a 9-point scale: 1: Very Poor/Irritating/Diﬃcult, 5: Neutral, 9: Excellent/Pleasing/Easy, and NA: Not Applicable. We organized different sections with the aim of evaluating interaction quality, usability, and overall user satisfaction across different functional areas.

The first area, User Interface and Navigation, evaluates the login process and the simplicity of navigating through the system. Users gave high ratings to the simplicity of entering credentials, the clarity of error messages, and the responsiveness of the login page. When assessing navigation, they evaluated the clarity of menus and options, the consistency of layout and flow, and the ease of transitioning between sections. The results show a consistently positive experience, with only minor differences among evaluators.

In the Query Execution area, we thoroughly examined the system’s ability to support different types of searches. Users rated the search for species by a selected metabolite highly, noting the ease of data entry and the clarity of the retrieved results. They considered the search for metabolites by a selected species particularly effective and useful for profiling purposes, while they consistently assigned the highest ratings to the search by genus or family for its clarity and relevance. When performing a search by expected *m*/*z*, users highlighted the accuracy of the entry fields, the clarity of the input interface, and the usefulness of the ‘Clear’ button. They judged the search by molecular formula and RT as highly intuitive, with well-defined default values that facilitated data entry. Finally, they found the search by fragments effective for supporting detailed analysis thanks to the completeness and relevance of the results. The third area, Data Entry and Updates, evaluates how users insert and modify metabolite data. Users emphasized the clarity of input fields—including ionization, fragments, and species—and praised the responsiveness of the validation process. They described updating existing entries as intuitive, noting that the system consistently provides clear and timely feedback after confirming changes.

The Spectrometry area evaluates the usability of tools for managing and analysing spectral data. Users highlighted the ease of file selection, the clarity of spectrum visualization, and the simplicity of peak identification. They also reported that entering associated information—such as metabolite name, RT, ionization type, and adducts—was straightforward and unambiguous, supporting an efficient workflow.

The Terminology and System Information area evaluates the layout and consistency of on-screen information, the terminology applied across the system, and the clarity of error and feedback messages. Users confirmed that the terminology remains consistent, while system messages are timely, precise, and informative, effectively guiding interaction, and error correction.

The evaluation of the last area, Learning, focuses on how easily users could become proficient with the system. Users found both basic and advanced functionalities accessible, reporting a short learning curve even during first-time use. They emphasized that the interface actively supports a smooth transition from initial exploration to effective operation.

The QUIS results demonstrate a high level of user satisfaction, with average ratings ranging between 8 and 9 for all evaluated areas. These findings suggest that the platform meets user needs, providing an intuitive, usable, and functionally robust tool for metabolomic analysis and profiling. In Table 1, we show an extract of the evaluation, while the complete QUIS items and scores can be found in Table A1 in the Appendix.

**Table 1:** QUIS results.

Aspect evaluated	Researcher 1	Researcher 2	Researcher 3
User Interface and Navigation			
Login and Accessibility			
Ease of entering credentials	9	8	9
Responsiveness of the login page	9	8	9
Query Execution			
Search for species by a selected metabolite			
Ease of inserting a metabolite	8	8	9
Search metabolites by molecular formula and retention time (RT)			
Clarity of the molecular formula input field	9	9	9
Data Entry and Updates			
Inserting a new metabolite			
Clarity of form fields	9	8	8
Spectrometry			
Spectrometry Tool Usability			
Ease of selecting a spectrum file	8	9	9
Ease of selecting the peaks of the displayed spectrum	8	9	9
Learning			
Learning to Operate the System			
Ease of getting started	9	7	8
Learning advanced features	8	8	8

## Discussion and conclusions

Despite the successful implementation and positive evaluation of the platform, this study presents some limitations that should be addressed in future developments. First, the user evaluation was conducted on a limited sample size of three domain experts from the NBFC project. While this provided high-quality qualitative feedback appropriate for a specialized tool, a larger-scale quantitative study involving external researchers would be necessary to statistically validate the usability results and the learning curve reported in the QUIS evaluation. Second, regarding the system architecture, the integration with MS instrumentation is currently semi-automated. The workflow relies on the generation of an Excel file by the instrument, which must then be manually uploaded by the researcher to the web platform. Future iterations of the system aim to develop direct API interfaces with the laboratory equipment to enable real-time data ingestion, thereby further reducing the risk of manual handling errors. Finally, the current database population is strictly focused on the core collection of ~700 species selected to represent the Italian plant families. While this fulfills the specific mandate of the NBFC project, extending the database schema to accommodate broader international biodiversity datasets would require further testing to ensure scalability and query performance.

To conclude and summarize, this work presents the development of a comprehensive web-based platform designed to manage the complexity of biodiversity and metabolomics data. First, we addressed the transition from non-standardized data storage to a structured system capable of ensuring integrity and reproducibility. Secondly, we adopted a process-oriented agile approach during the requirement analysis, which is a key strength of our approach. With this technique, we ensured that the platform accurately reflects the real-world laboratory practices. Thirdly, the database integration with external resources significantly enriched the analysis of experimental data, allowing for the automatic retrieval of chemical structures and taxonomic hierarchies. Furthermore, another key strength of our work is the implementation of the spectrometry tool for the automatic analysis of spectral data. Finally, the user evaluation phase with positive feedback confirms that the platform meets the researchers’ needs.

## Data Availability

This paper proposes an original agile- and process-oriented methodology, for the design and implementation of a web-based platform specifically developed to support the valorization of biodiversity research, within the activities of the National Biodiversity Future Center (NBFC). The data used in this study serve exclusively as an illustrative application of the proposed technical framework. This is the repository https://github.com/BeatriceAmico01/NBFC-project of the project, which includes data and code.

## Appendix 1 QUIS complete results

**Table A1:** QUIS evaluation.

Evaluated aspect	Researcher 1	Researcher 2	Researcher 3
User Interface and Navigation			
Login and Accessibility			
Ease of entering credentials	9	8	9
Responsiveness of the login page	9	8	9
Clarity of error messages (e.g. incorrect password)	9	8	9
Navigation			
Ease of navigating between pages	8	8	9
Clarity of menus and options	9	6	9
Consistency in layout and navigation flow	9	8	9
Query Execution			
Search for species by a selected metabolite			
Ease of inserting a metabolite	8	8	9
Relevance and clarity of the information presented in the obtained results	9	8	8
Ease and usefulness of the query for the profiling purpose	8	8	9
Search for metabolites by a selected species			
Ease of inserting the species	9	9	9
Relevance and clarity of the information in the results	9	9	8
Ease and usefulness for profiling purposes	8	9	9
Search for metabolites by a selected genus or family			
Ease of inserting the genus/family	9	9	9
Relevance and clarity of the information in the results	9	9	9
Ease and usefulness for profiling purposes	9	9	9
Search for metabolites by an expected *m*/*z*			
Clarity of the expected *m*/*z* range input field	9	9	9
Relevance and clarity of the information in the results	9	9	9
Clarity of the functionality of the ‘Clear’ button	8	9	9
Ease and usefulness for profiling purposes	9	9	9
Search metabolites by molecular formula and retention time (RT)			
Clarity of the molecular formula input field	9	9	9
Clarity of the retention time input field	9	9	8
Clarity of the RT range input field with default value	9	9	9
Relevance and clarity of the information in the results	9	9	9
Clarity of the functionality of the ‘Clear’ button	9	9	9
Ease and usefulness for profiling purposes	9	9	9
Search for metabolites by fragments			
Clarity of the ionization type input field	9	9	9
Clarity of the fragment range input field	9	9	8
Clarity of the fragment input field (max 3 fragments)	9	9	9
Relevance and clarity of the information in the results	9	9	9
Clarity of the functionality of the ‘Clear’ button	9	9	9
Ease and usefulness for profiling purposes	9	9	9
Data Entry and Updates			
Inserting a new metabolite			
Clarity of form fields	9	8	8
Ease of inputting data (e.g. selecting species, adding fragments)	8	9	8
Clarity of error message when a mandatory field is missing	9	9	9
Responsiveness of the form validation process	9	9	9
Updating a metabolite			
Clarity of the update interface	8	9	9
Ease of making changes to existing data	8	9	9
Intuitiveness of the editable fields	9	9	9
Quality of the feedback after submitting updates	8	9	9
Spectrometry			
Spectrometry Tool Usability			
Ease of selecting a spectrum file	8	9	9
Ease of selecting the peaks of the displayed spectrum	8	9	9
Clarity of the displayed spectrum	8	9	9
Ease of inputting data: metabolite name, retention time, ionization, adduct, setting information	8	9	9
Ease in validating the form	8	9	9
Terminology and System Information			
Screen Layout and Information Display			
Amount of displayed information on screen	8	9	8
Arrangement of information on screen	8	9	7
Consistent arrangement of information	8	9	9
Use of Terms Throughout the System			
Screen headings	8	9	9
Messages (feedback) on screen	9	8	9
Terms on the screen (item labels)	9	9	8
Location of messages on the screen	9	9	9
Instructions for commands or choices	9	8	9
Instructions for Correcting Errors			
System keeps you informed about actions	8	9	9
Performing an operation leads to predictable results	9	9	9
User can control the amount of feedback	8	9	9
Error Messages			
Error messages clarify the problem	9	8	9
Learning			
Learning to Operate the System			
Ease of getting started	9	7	8
Learning advanced features	8	8	8
Time to learn to use the system at first attempt	8	9	8
Overall Use			
Overall System Experience			
Overall ease of use	9	9	8
Visual appeal of the application interface	9	8	7
Speed and responsiveness of the application	8	8	9
Usefulness of the application for your work	8	9	8
